# Triglyceride glucose-waist circumference, a novel and effective predictor of diabetes in first-degree relatives of type 2 diabetes patients: cross-sectional and prospective cohort study

**DOI:** 10.1186/s12967-016-1020-8

**Published:** 2016-09-07

**Authors:** Shuang Zheng, Sheng Shi, Xingxing Ren, Tingting Han, Yangxue Li, Yawen Chen, Wei Liu, Peter C. Hou, Yaomin Hu

**Affiliations:** 1 Department of Endocrinology, Renji Hospital, School of Medicine, Shanghai Jiaotong University, No.160 Pujian Road, Shanghai, 200127 China; 2 Department of Orthopedic Surgery, Renji Hospital, School of Medicine, Shanghai Jiaotong University, No.160 Pujian Road, Shanghai, 200127 China; 3 Department of Emergency Medicine, Brigham and Women’s Hospital and Harvard Medical School, Boston, MA USA

**Keywords:** Type 2 diabetes mellitus, First-degree relative, Triglyceride glucose index, Visceral adiposity index, TyG-WC

## Abstract

**Background:**

Body mass index (BMI), waist circumference (WC), visceral adiposity index (VAI), triglyceride glucose index (TyG), TyG-BMI, and TyG-WC have been reported as markers of insulin resistance or type 2 diabetes mellitus (T2DM). However, little is known about the associations between the aforementioned markers and the risk of prediabetes and diabetes in first-degree relatives (FDRs) of T2DM patients.

**Methods:**

1544 FDRs of T2DM patients (635 men and 909 women) were enrolled in the initial cross-sectional study and all of them finished corresponding examinations. Logistic regression analysis and receiver operating characteristic (ROC) curve were used to compare and identify the associations of the six parameters (BMI, WC, VAI, TyG, TyG-BMI and TyG-WC) with the prevalence of prediabetes and diabetes. Subsequently, 452 of them were followed-up for an average of 5 years. Cox proportional hazard regression model was applied to confirm the predictive value of the optimal marker.

**Results:**

Among the indices, TyG-WC was more strongly associated with the prevalence of prediabetes and diabetes. Compared with participants in the lowest quartile of TyG-WC, the adjusted odds ratio and 95 % CIs for prediabetes and diabetes was 11.19 (7.62–16.42) for those in the top quartile of TyG-WC. Moreover, the largest AUC was also observed in TyG-WC (0.765, 95 % CIs 0.741–0.789, P < 0.001). The robust predictive value of TyG-WC was further confirmed in the follow-up study (HR: 7.13, 95 % CIs 3.41–14.90, P < 0.001).

**Conclusions:**

TyG-WC is a novel and clinically effective marker for early identifying the risks of prediabetes and diabetes in FDRs of T2DM patients.

**Electronic supplementary material:**

The online version of this article (doi:10.1186/s12967-016-1020-8) contains supplementary material, which is available to authorized users.

## Background

The occurrence rate of type 2 diabetes mellitus (T2DM) is quite astonishing worldwide, of which is a major risk factor for cardiovascular disease and even premature mortality [1]. Thus, it is of utmost significance to early identify and treat subjects at high risk of developing T2DM, though the unclear etiologies and pathological process of it. Familial clustering phenomenon of T2DM may back the genetic susceptibility to T2DM. Ma et al. [2] demonstrated that first-degree relatives (FDRs) of patients with T2DM may have a higher prevalence of diabetes than those without a family history of T2DM (26.6 versus 9.2 %). Therefore, it is more important to early determine the susceptible population vulnerable to T2DM via simple and effective diagnostic tools, considering the enormous population of FDRs.

Previous literature indicated that several effective and inexpensive variables, ranging from simple anthropometric measures to more complex models, are closely related to insulin resistance (IR) or diabetes. Body mass index (BMI) and waist circumference (WC), two clinical indices for body fat assessment, are commonly used for detecting prediabetes and diabetes risk [3, 4]. Moreover, visceral adiposity index (VAI), a mathematical model based on BMI, WC, triglyceride (TG) and high-density lipoprotein cholesterol (HDL-C), is a more effective tool for prediabetes and diabetes prediction [5, 6]. In addition, triglyceride glucose index (TyG) as well as TyG-related indicators (TyG-BMI and TyG-WC) have been reported as excellent surrogate markers of IR, which is deemed to be the vital pathological mechanism of T2DM [7, 8]. To our knowledge, little is known about the accuracy and predictability of these indicators in suffering prediabetes and diabetes in FDRs of T2DM patients.

The objectives of the present study were to investigate the corresponding associations of the aforementioned indicators with the prevalence of prediabetes and diabetes in FDRs of T2DM patients and identify the excellent one firstly. Subsequently, a follow-up study was conducted to evaluate the incidence of diabetes in this population and further assess the performance of the optimal indicator in predicting the risk of T2DM.

## Methods

### Participants

Stratified random sampling was performed to select T2DM patients from the database of Renji hospital from January 1995 to 2005. The family of each randomly selected subject were contacted by telephone or door-to-door visit. Only one of the FDRs (including parents, children and full siblings) of each T2DM patient was randomly selected and invited to our study from September 2005 to August 2009. A total of 2392 FDRs of these T2DM patients were invited to the survey. After excluding ineligible subjects, 2018 FDRs were recruited to the study and finished structured questionnaires on their first visit. Next, 474 subjects were further excluded according to the exclusion criteria including self-reported diabetes diagnosis and/or regular diabetic medication use, less than 18 or more than 90 years old, pregnant, chronic renal or hepatic failure, cancer, taking regular medication for dyslipidemia and/or hypertension. Finally, 1544 subjects (635 men and 909 women) were enrolled in the cross-sectional study.

**Fig. 1:**
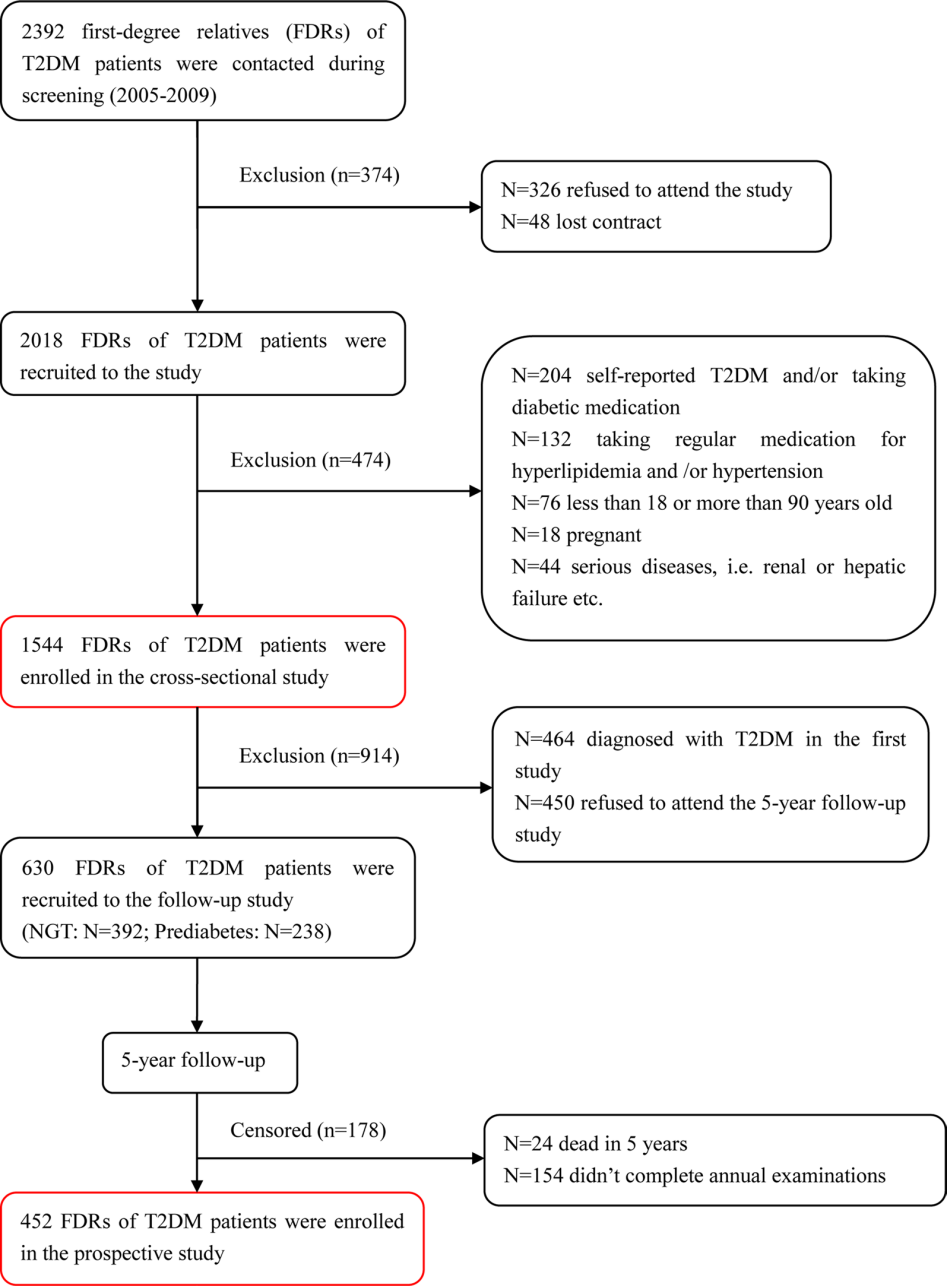
Trial profile. *OGTT* oral glucose tolerance test, *FDR* first degree relative, *NGT* normal glucose tolerance

To further test whether the optimal marker identified through cross-sectional study is useful for predicting incident diabetes, we conducted a 5-year prospective cohort study including FDRs of T2DM patients diagnosed with NGT or prediabetes in the initial study. After excluding ineligible participants, 452 of the 1544 FDRs completed the annual examinations with the average duration of 5 years (Fig. 1).

The study protocol was in compliance with the declaration of Helsinki and approved by the Ethical Committee of Renji Hospital, School of Medicine, Shanghai Jiaotong University. Written informed consents were signed from all participants included in the study.

### Measurements

Body height, weight, WC and blood pressure (BP) were measured by trained survey personnel. Both height and weight measurements were taken in light clothing without shoes. The smallest abdominal circumference was measured as WC, which was taken twice and the mean value was recorded. Blood pressure was measured three times in each subject on the right arm after 5 min resting in a sitting position, and the mean value was recorded.

### Laboratory analysis

Each participant received a 75 g OGTT after at least 10 h of overnight fasting. Blood samples were collected at 0, 30, 60, 120 and 180 min after the glucose load. Plasma glucose levels were measured using the glucose oxidase method. Serum insulin levels were obtained using a bio-antibody technique (Linco, St Louis, MO, USA). Serum lipid profiles were tested with an automated biochemical instrument by radioimmunoassay (RIA) based on the double-antibody technique (DPC, Los Angeles, CA, USA). HbA1c was measured by the high-performance liquid chromatography (HPLC) method with a BIO-RAD analyzer (Bio-Rad Variant II; Bio-Rad Laboratories, Hercules, CA, USA).

### Diagnostic criteria and definition

The 1999 World Health Organization (WHO) diagnostic criteria for T2DM was adopted [9].

Normal glucose tolerance (NGT) was defined as fasting plasma glucose <6.1 mmol/l and 2-h plasma glucose <7.8 mmol/l. Prediabetes includes isolated impaired fasting glucose (IFG), isolated impaired glucose tolerance (IGT) and combined glucose intolerance (CGI). IFG was defined as fasting plasma glucose between 6.1 and 7.0 mmol/l and 2-h plasma glucose <7.8 mmol/l; IGT was defined as fasting plasma glucose <6.1 mmol/l and 2-h plasma glucose between 7.8 and 11.1 mmol/l; CGI was defined as fasting plasma glucose between 6.1 and 7.0 mmol/l and 2-h plasma glucose between 7.8 and 11.1 mmol/l. Diabetes mellitus (DM) was defined as fasting plasma glucose ≥7.0 mmol/l and/or 2-h plasma glucose ≥11.1 mmol/l.

BMI was calculated as the body weight (kg) divided by the square of body height (m^2^). VAI and TyG were calculated using the former formula [10]. VAI: Men: [WC/(39.68 + 1.88 × BMI)] × (TG/1.03) × (1.31/HDL); Women: [WC/(36.58 + 1.89 × BMI)] × (TG/0.81) × (1.52/HDL), where both TG and HDL levels are expressed in mmol/L. The TyG index: Ln [TG (mg/dl) × FPG (mg/dl)/2]. TyG-BMI: TyG index × BMI. TyG-WC: TyG index × WC (cm).

Incidence was calculated as the number of T2DM cases per 100 person years of follow-up starting from the date of finishing the initial examination in 2005–2009 to the occurrence of diabetes or the final follow-up visit in the 5th year.

### Statistical analysis

All data were analyzed using SPSS version 17.0 for Windows (SPSS, Chicago, IL, USA). Continuous data were shown as medians and interquartile ranges (IQR) by virtue of the skewed distribution and compared utilizing Kruskal–Wallis H test or Mann–Whitney U test. Adjusted means were calculated and compared with general linear models. Categorical variables were expressed as percentages and compared with Chi square test. Multinomial logistic regression was conducted to determine the correlations between different factors and the risk of prediabetes and diabetes after controlling potential confounding factors. For each indicator, odds ratios and 95 % CIs of quartiles 2–4 were calculated and compared using quartile 1 as the reference. Receiver operating characteristic (ROC) curves were applied to compare the relative diagnostic strengths of these indicators for correctly discriminating prediabetes and diabetes. The area under the ROC curve (AUC) was utilized to quantify the overall diagnostic accuracy. Indicator with the largest AUC was considered as the best marker. The cutoff point of the optimal indictor was calculated according to Youden Index and the corresponding sensitivity, specificity, positive and negative predictive values were further assessed in the cohort study. Cox proportional hazard regression was taken to evaluate the predictive power of the optimal marker for incident diabetes after adjusting for confounding factors. Probability value less than 0.05 was considered statistically significant.

## Results

### Baseline characteristics

A total of 1544 participants were enrolled in the cross-sectional study, including 657 with NGT, 423 with prediabetes and 464 with previously undiagnosed diabetes. Baseline characteristics of participants, stratified by glucose tolerance status, were presented in Table 1. The median ages of subjects with NGT, prediabetes and diabetes were 47.0, 52.0 and 59.0 years old, respectively (P < 0.05). After adjusting for age, subjects with prediabetes and diabetes had higher levels of blood pressure, VAI, BMI, WC, TyG, TyG-BMI, TyG-WC, TG, LDL-C and lower levels of HDL-C than those with NGT.

**Table 1:** Baseline characteristics of study population

Characteristics	NGT	Prediabetes	Diabetes
Number	657	423	464
Sex (M/F)	256/401	174/249	205/259
Age (years)	47.0 (36.0, 54.0)	52.0 (44.0, 58.0)^a^	59.0 (50.0, 65.0)^a, b^
SBP (mmHg)	121.0 (119.0, 122.0)	126.0 (124.0, 127.0)^a^	129.0 (127.0, 131.0)^a, b^
DBP (mmHg)	76.0 (75.0, 77.0)	79.0 (78.0, 80.0)^a^	79.0 (78.0, 80.0)^a^
BMI (kg/m2)	24.39 (24.03, 24.75)	25.12 (24.68, 25.55)^a^	25.26 (24.81, 25.70)^a^
WC (cm)	83.0 (82.1, 83.9)	88.4 (87.3, 89.5)^a^	92.2 (91.1, 93.3)^a, b^
VAI	1.70 (1.53, 1.87)	2.39 (2.19, 2.59)^a^	2.82 (2.62, 3.03)^a, b^
TyG	8.43 (8.38, 8.48)	8.81 (8.75, 8.87)^a^	9.21 (9.15, 9.27)^a, b^
TyG-BMI	206.47 (202.86, 210.08)	222.05 (217.69, 226.42)^a^	233.45 (228.99, 237.91)^a, b^
TyG-WC	702.07 (692.13, 712.00)	780.36 (768.35, 792.37)^a^	851.94 (839.66, 864.22)^a, b^
TG (mmol/L)	1.33 (1.23, 1.43)	1.71 (1.59, 1.82)^a^	1.94 (1.82, 2.07)^a, b^
TC (mmol/L)	4.92 (4.83, 5.00)	5.01 (4.91, 5.11)	5.09 (4.98, 5.19)^a^
HDL-C (mmol/L)	1.47 (1.44, 1.50)	1.31 (1.27, 1.35)^a^	1.31 (1.27, 1.34)^a^
LDL-C (mmol/L)	2.93 (2.87, 2.99)	3.03 (2.96, 3.10)^a^	3.06 (2.98, 3.13)^a^
FPG (mmol/L)	5.10 (4.97, 5.22)	5.74 (5.59, 5.89)^a^	8.65 (8.50, 8.81)^a, b^
2hPG (mmol/L)	6.06 (5.83, 6.29)	8.65 (8.38, 8.93)^a^	17.11 (16.83, 17.39)^a, b^
FINS (μU/ml)	9.23 (8.55, 9.92)	11.19 (10.36, 12.03)^a^	12.58 (11.73, 13.43)^a, b^
2hINS (μU/ml)	38.70 (36.09, 41.31)	65.52 (62.36, 68.68)^a^	43.02 (39.80, 46.25)^b^
HbA1c (%)	5.50 (5.42, 5.58)	5.75 (5.65, 5.84)^a^	7.36 (7.27, 7.46)^a, b^

Data were expressed as median (Interquartile range 25–75 %)

Comparisons among NGT, Prediabetes and Diabetes groups were performed after adjusting for age

*SBP* systolic blood pressure, *DBP* diastolic blood pressure, *BMI* body mass index, *WC* waist circumference, *VAI* visceral adiposity index, *TyG* triglyceride glucose index, *TyG-BMI* combined TyG and BMI, *TyG-WC* combined TyG and WC, *TG* triglyceride, *TC* total cholesterol, *HDL-C* high density lipoprotein cholesterol, *LDL-C* low density lipoprotein cholesterol, *FPG* fasting plasma glucose, *2hPG* 2 h postload plasma glucose, *FINS* fasting serum insulin, *2hINS* 2 h postload serum insulin, *HbA1c* glycated hemoglobin A1c

^a^P < 0.05 versus NGT group

^b^P < 0.05 versus Prediabetes group

### Associations of indicators with prediabetes and diabetes risk

The ORs and 95 % CIs for prediabetes and/or diabetes were progressively increased across quartiles of each index after adjusting for age, sex, SBP and DBP (Table 2). After direct comparison, TyG-WC presented the highest ORs and 95 % CIs for prediabetes and diabetes, reaching 11.19 (95 % CIs 7.62–16.42) for the top quartile as compared with the bottom quartile (P < 0.001), followed by TyG index (Q4 11.04, 95 % CIs 7.57–16.09) and WC (Q4 5.65, 95 % CIs 3.97–8.04).

**Table 2:** Adjusted odds ratios (OR) for prediabetes and diabetes in quartiles of each index

Parameters	Prediabetes OR (95 % CI)	P value	Diabetes OR (95 % CI)	P value	Prediabetes + diabetes OR (95 % CI)	P value
*VAI*						
Q1 (≤0.981)	1	–	1	–	1	–
Q2 (−1.630)	1.26 (0.86–1.83)	0.239	1.31 (0.89–1.93)	0.172	1.28 (0.94–1.74)	0.123
Q3 (−2.633)	3.16 (2.19–4.57)	<0.001	2.28 (1.54–3.38)	<0.001	2.72 (1.98–3.75)	<0.001
Q4 (≥2.634)	4.07 (2.76–6.00)	<0.001	4.57 (3.07–6.81)	<0.001	4.23 (3.03–5.90)	<0.001
*BMI*						
Q1 (≤22.49)	1	–	1	–	1	–
Q2 (−24.61)	1.40 (0.97–2.01)	0.069	2.05 (1.38–3.03)	<0.001	1.64 (1.21–2.24)	0.002
Q3 (−26.82)	1.41 (0.99–2.02)	0.057	2.09 (1.40–3.10)	<0.001	1.66 (1.21–2.27)	0.002
Q4 (≥26.83)	1.75 (1.21–2.53)	0.003	2.45 (1.63–3.68)	<0.001	2.03 (1.46– 2.80)	<0.001
*WC*						
Q1 (≤79.0)	1	–	1	–	1	–
Q2 (−87.0)	1.64 (1.16–2.33)	0.005	4.64 (2.85–7.56)	<0.001	2.27 (1.66–3.10)	<0.001
Q3 (−94.0)	2.72 (1.88–3.95)	<0.001	11.16 (6.83–18.23)	<0.001	4.50 (3.22–6.28)	<0.001
Q4 (≥94.1)	3.05 (2.05–4.52)	<0.001	15.36 (9.27–25.43)	<0.001	5.65 (3.97–8.04)	<0.001
*TyG*						
Q1 (≤8.269)	1	–	1	–	1	–
Q2 (−8.724)	2.07 (1.45–2.97)	<0.001	2.69 (1.68–4.33)	<0.001	2.21 (1.60–3.04)	<0.001
Q3 (−9.186)	3.19 (2.19–4.64)	<0.001	6.66 (4.18–10.63)	<0.001	4.16 (2.98–5.80)	<0.001
Q4 (≥9.187)	6.54 (4.29–9.97)	<0.001	23.04 (14.03–37.82)	<0.001	11.04 (7.57–16.09)	<0.001
*TyG-BMI*						
Q1 (≤190.27)	1	–	1	–	1	–
Q2 (−214.53)	1.87 (1.29–2.72)	0.001	2.41 (1.56–3.72)	<0.001	2.06 (1.50–2.81)	<0.001
Q3 (−243.30)	1.92 (1.35–2.73)	<0.001	3.79 (2.47–5.82)	<0.001	2.50 (1.81–3.46)	<0.001
Q4 (≥243.31)	3.52 (2.37–5.23)	<0.001	9.04 (5.76–14.18)	<0.001	5.27 (3.72–7.47)	<0.001
*TyG-WC*						
Q1 (≤668.00)	1	–	1	–	1	–
Q2 (−758.52)	1.67 (1.17–2.38)	0.004	4.39 (2.56–7.50)	<0.001	2.19 (1.59–3.02)	<0.001
Q3 (−849.62)	4.12 (2.83–6.01)	<0.001	15.33 (8.92–26.33)	<0.001	6.25 (4.42–8.84)	<0.001
Q4 (≥849.63)	4.99 (3.25–7.66)	<0.001	38.69 (22.01–68.02)	<0.001	11.19 (7.62–16.42)	<0.001

All indices were divided into quartiles and examined by multinomial logistic analysis. P value was adjusted for age, sex, systolic blood pressure and diastolic blood pressure

*VAI* visceral adiposity index, *BMI* body mass index, *WC* waist circumference, *TyG* triglyceride glucose index, *TyG-BMI* combined TyG and BMI, *TyG-WC* combined TyG and WC

**Fig. 2:**
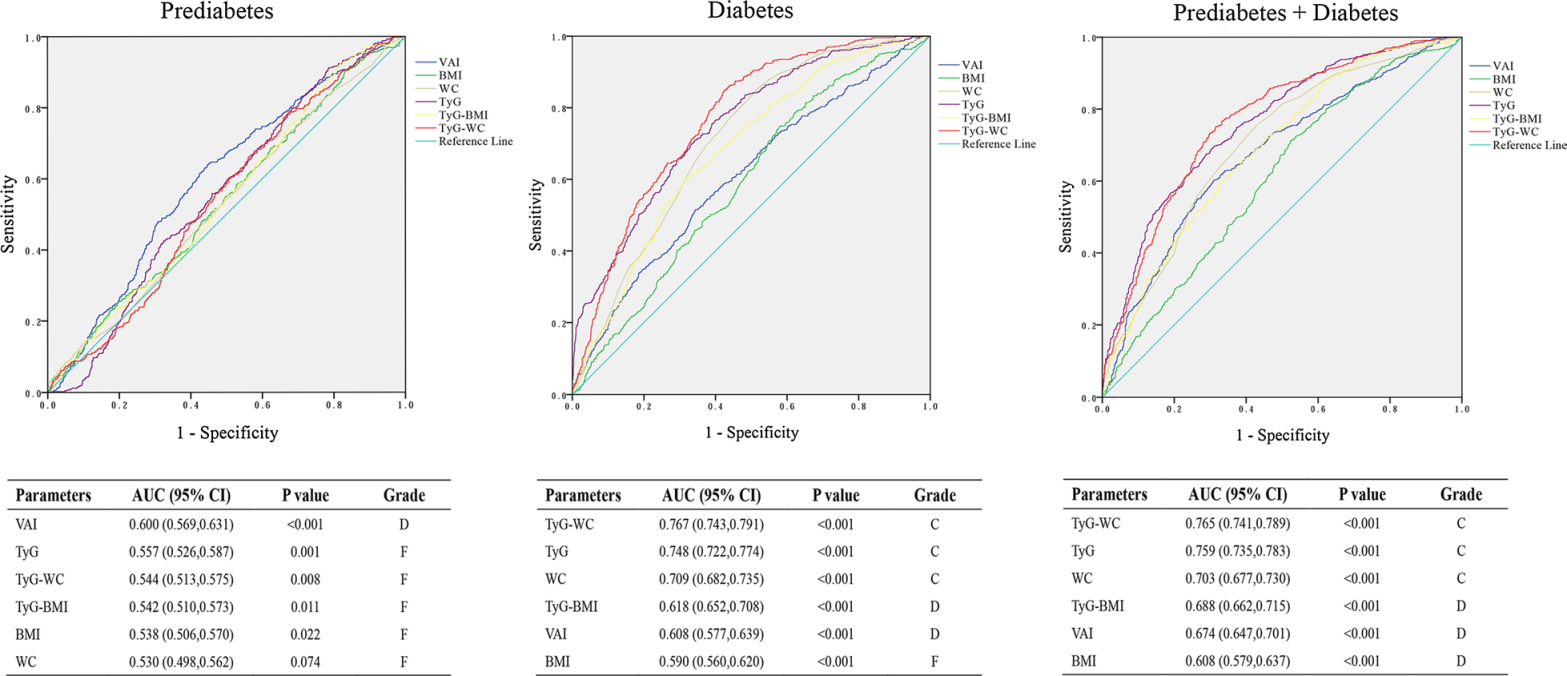
Receiver operating characteristic (ROC) curve analysis of each index. Area under the ROC curve (AUC) and 95 % CIs of each index was shown in tables below. *VAI* visceral adiposity index, *BMI* body mass index, *WC* waist circumference, *TyG* triglyceride glucose index, *TyG-BMI* combined TyG and BMI, *TyG-WC* combined TyG and WC. Grade: 0.90–1 = excellent (A), 0.80–0.90 = good (B), 0.70–0.80 = fair (C), 0.60–0.70 = poor (D) and 0.50–0.60 = fail (F)

The results of ROC analyses and AUCs with their corresponding 95 % CIs for VAI, BMI, WC, TyG, TyG-BMI and TyG-WC were shown in Fig. 2. For prediabetes, the largest AUC was observed in VAI (AUC = 0.600, 95 % CIs 0.569–0.631, Grade: D), followed by TyG (AUC = 0.557, 95 % CIs 0.526–0.587, Grade: F) and TyG-WC (AUC = 0.544, 95 % CIs 0.513–0.575, Grade: F). For diabetes, the largest AUC was showed in TyG-WC (AUC = 0.767, 95 % CIs 0.743–0.791, Grade: C), followed by TyG (AUC = 0.748, 95 % CIs 0.722–0.774, Grade: C) and WC (AUC = 0.709, 95 % CIs: 0.682–0.735, Grade: C). For mixed prediabetes and diabetes, the largest AUC was also showed in TyG-WC (AUC = 0.765, 95 % CIs 0.741–0.789, Grade: C), followed by TyG (AUC = 0.759, 95 % CIs 0.735–0.783, Grade: C) and WC (AUC = 0.703, 95 % CIs 0.677–0.730, Grade: C). Taking the odds ratio and the AUC value into consideration, TyG-WC may be regarded as an optimal marker for predicting prediabetes and diabetes in those participants.

### Clinical outcomes at the final follow-up

Data from 184 men and 268 women with a median age at baseline of 51.0 and 48.0 years respectively were observed for an average of 5 years (4.62 ± 0.99). During the 2013 person-years of follow-up, 75 of the 452 participants were identified as newly occurred diabetes patients and the total incidence of diabetes was 3.7 per 100 person-years. When stratified by quartiles of TyG-WC, the incidences of diabetes, from Quartile1 to 4, were 1.2, 2.1, 5.1 and 9.6 per 100 person-years, respectively. In addition, the cumulative rates of incident diabetes from Q1 to Q4 were 5.9, 9.8, 21.9 and 35.9 %, respectively (Table 3).

**Table 3:** Incidence of diabetes according to TyG-WC quartiles

TyG-WC	Subjects N	New DM N	Person-years	DM Incidence/100 person-years	Cumulative incidence of DM (%)
Q1 (≤668.00)	153	9	740	1.2	5.9
Q2 (−758.52)	102	10	477	2.1	9.8
Q3 (−849.62)	105	23	451	5.1	21.9
Q4 (≥849.63)	92	33	345	9.6	35.9

*DM* diabetes mellitus, *TyG-WC* triglyceride glucose-waist circumference index

### The predictive value of TyG-WC

As compared to individuals with the lowest TyG-WC (Table 4), those who had the highest TyG-WC were at 7.13-fold risk of diabetes (95 % CIs 3.41–14.90). The positive trend between TyG-WC level and diabetes risk was attenuated but still remarkable after adjusting for age, sex, SBP, DBP, TC and LDL-C (HR: 3.69, 95 % CIs 1.65–8.28). Additionally, according to the results of ROC curve and the Youden Index, the optimal cutoff point of TyG-WC was 760.06, with the sensitivity of 74.7 % and the specificity of 63.1 %. Meanwhile, the positive and negative predictive values at this point were 28.7 and 92.6 %, respectively.

**Table 4:** Hazard Ratio (95 % CI) of diabetes risk according to TyG-WC quartiles

TyG-WC	Crude	Model 1	Model 2	Model 3
Q1 (≤668.00)	1 (referent)	1 (referent)	1 (referent)	1 (referent)
Q2 (−758.52)	1.70 (0.69–4.19)	1.58 (0.64–3.92)	1.33 (0.53–3.30)	1.21 (0.48–3.05)
Q3 (−849.62)	4.01 (1.86–8.66)^***^	3.55 (1.60–7.88)^**^	2.71 (1.21–6.05)^*^	2.34 (1.01–5.42)^*^
Q4 (≥849.63)	7.13 (3.41–14.90)^***^	6.41 (2.96–13.90)^***^	4.87 (2.23–10.65)^***^	3.69 (1.65–8.28)^**^

*Model 1* adjusted for age and sex

*Model 2* adjusted for Model 1 added SBP, DBP

*Model 3* adjusted for Model 2 added TC, LDL-C

* P < 0.05

** P < 0.01

*** P < 0.001

## Discussion

In the cross-sectional study, we directly compared six parameters (BMI, WC, VAI, TyG, TyG-BMI and TyG-WC) as predictors of prediabetes and diabetes in FDRs of T2DM patients. Overall, we found that TyG-WC outperformed other predictors with a higher OR and a larger AUC. Moreover, in the prospective study, we observed that subjects in the highest quartile of TyG-WC had 3.7-fold risk of diabetes for those in the lowest quartile even after the adjustment of potential compounders, which indicated that TyG-WC was an independent predictor of diabetes in FDRs of T2DM patients.

Previous studies have indicated that both genetic and environmental factors contribute to the development of diabetes [11–13]. Of note, the prevalence of the multifactorial disease and potential population of FDRs of T2DM patients are increasing obviously with the change of lifestyle [14]. FDRs of T2DM patients are regarded as high-risk diabetic populations, considering the genetic predisposition and the similar lifestyle [15, 16]. In the current study, the crude prevalence of diabetes in FDRs was 30.1 % and the age-standardized prevalence was 15.6 %, which was higher than the national prevalence of diabetes in China (9.7 %) [17]. Furthermore, Du et al. [18] demonstrated that the prevalence of diabetes was independently associated with an increasing family history risk level. Hence, more attention to FDRs of T2DM patients should be paid in the clinical diagnosis and treatment of diabetes in an early stage, though the contribution of genetic factors to the pathological development of diabetes remains obscure.

The strong relationship between obesity and diabetes has been mentioned in many studies [19–21]. Oti et al. [22] found that obesity is closely associated with high blood glucose. Matsuda et al. [23] maintained that adipose tissue is the main source of reactive oxygen species, which may contribute to a variety of metabolic problems, including obesity-associated IR and T2DM. As simple, cheap and noninvasive anthropometric parameters, BMI and WC are commonly adopted as useful indicators of obesity and metabolic risk. However, recent studies indicated that some populations show unexpected metabolic profiles that deviate from the typical dose-response relationship between BMI and metabolic disturbances [24–26]. In the current study, we also found the association between BMI and abnormal glucose metabolism was weaker than that of WC when considering the lower odds ratios and AUCs of BMI. These results may be explained by the different roles of BMI and WC in the evaluation of adiposity status [27]. BMI, a measure of body fat based on weight and height, stands for general obesity, while WC, a measure of abdominal fat, stands for central obesity. The National Cholesterol Education Program-Adult Treatment Panel-III suggested that central obesity is an independent risk factor for T2DM, and measuring WC is an inexpensive tool to screen risk of diabetes [28]. Therefore, WC may be more effective than BMI. However, WC cannot sufficiently discriminate between visceral and subcutaneous fat. Accumulating evidence has demonstrated that visceral adipose tissue plays more critical roles in the development of insulin resistance and diabetes than subcutaneous fat. Molecular mechanisms responsible for the differences are still under discussion. It has been suggested that visceral fat produces more free fatty acid than subcutaneous fat, thus increases the risk of IR and diabetes [29]. Moreover, visceral adipose secretes various inflammatory cytokines and adipokines, which may also promote the occurrence of IR and diabetes [30, 31].

Besides obesity, increased FPG levels have also been demonstrated as an independent risk factor for developing T2DM [32–34]. Moreover, elevated TG levels over time also enhance the risk of developing diabetes in various populations [35–38]. Additionally, Guerrero-Romero et al. suggested that, TyG index, the product of FPG and TG, could be a surrogate index of insulin resistance due to its high sensitivity similar to euglycemic-hyperinsulinemic clamp test [6]. Meanwhile, it is also proposed that TyG index is a valuable marker for predicting the risk of future diabetes in both men and women [39]. Given that insulin resistance is the core pathological mechanism of T2DM and always occurs before the diagnosis of T2DM [40, 41], surrogate indices of insulin resistance might aid in the prediction of incident diabetes. In our study, we found TyG-WC, the combination of adiposity status and TyG, was a better marker for early predicting the risk of prediabetes and diabetes. The superiority of TyG-WC might be achieved as TG, FPG and obesity are well validated for their roles in IR and the development of diabetes. These results also support that both glucotoxicity and lipotoxicity play crucial roles in the pathogenesis of diabetes.

The visceral adiposity index (VAI), a mathematical model based on BMI, WC, TG and HDL-C, is another predictor of prediabetes and diabetes demonstrated by several research, although the relationship might differ by ethnicity [42, 43]. In our study, we found the association between VAI and diabetes was weaker than that of TyG related parameters. However, it was noteworthy that VAI was better correlated with prediabetes than with diabetes, which was commensurate with the study of Yang et al. [44]. Underlying mechanisms are still unclear. Possible explanation might be that subjects with prediabetes have better glucose regulation than those with diabetes, so the effect of glucotoxicity was slight in this stage. Thus, VAI, an index stands for the condition of obesity and lipid levels, is closely related to prediabetes while TyG and related parameters are well associated with diabetes.

In the follow-up study, we further evaluated the clinical outcomes of participants and confirmed the predictive value of TyG-WC. Notably, the incidences of diabetes were significantly increased in sequence of quartiles of TyG-WC. Furthermore, compared with participants in the highest quartile of TyG-WC value, the hazard ratio of incident diabetes was more than threefold for those in the lowest quartile after adjusting for age, gender, blood pressure and other potential compounders, which demonstrated the close association between TyG-WC and diabetes risk.

Several limitations may exist in this study. First, the results might have potential bias due to the single-center design. Second, some potential bias from socio-economic background and general diet intake were not well controlled. Third, the number of participants in the follow-up study was relative small. Fourth, the results were obtained from FDRs of diabetes patients, and further investigations were required in other populations.

## Conclusions

TyG-WC is a valuable marker for predicting the risk of prediabetes and diabetes in FDRs of T2DM patients. Because it can be easily calculated from routine laboratory data, we suggest the possibility of applying this index in risk assessment in real clinical practice or epidemiologic survey.

## Additional file

10.1186/s12967-016-1020-8 Clinical dataset.

## Abbreviations

BMI: body mass index
CGI: combined glucose intolerance
HDL-C: high density lipoprotein cholesterol
IFG: impaired fasting glucose
IGT: impaired glucose tolerance
LDL-C: low density lipoprotein cholesterol
NGT: normal glucose tolerance
OGTT: oral glucose tolerance test
TC: total cholesterol
TG: triglyceride
TyG: triglyceride glucose index
VAI: visceral adiposity index
WC: waist circumference
